# Brain Hemispheres Swap Dominance for Processing Semantically Meaningful Pitch

**DOI:** 10.3389/fnhum.2021.621677

**Published:** 2021-02-03

**Authors:** Xiao-Dong Wang, Hong Xu, Zhen Yuan, Hao Luo, Ming Wang, Hua-Wei Li, Lin Chen

**Affiliations:** ^1^Faculty of Psychology, Southwest University, Chongqing, China; ^2^Auditory Research Laboratory, School of Life Sciences, University of Science and Technology of China, Hefei, China; ^3^Division of Psychology, School of Social Sciences, Nanyang Technological University, Singapore, Singapore; ^4^Bioimaging Core, Faculty of Health Sciences, University of Macau, Macau, China; ^5^Department of Otolaryngology-Head and Neck Surgery, Wayne State University School of Medicine, Detroit, MI, United States; ^6^Affiliated Eye and ENT Hospital, Fudan University, Shanghai, China

**Keywords:** brain laterality, lexical tone, mismatch negativity, reaction time, auditory processing

## Abstract

The question of what determines brain laterality for auditory cognitive processing is unresolved. Here, we demonstrate a swap of hemisphere dominance from right to left during semantic interpretation of Chinese lexical tones in native speakers using simultaneously recorded mismatch negativity response and behavioral reaction time during dichotic listening judgment. The mismatch negativity, which is a brain wave response and indexes auditory processing at an early stage, indicated right hemisphere dominance. In contrast, the behavioral reaction time, which reflects auditory processing at a later stage, indicated a right ear listening advantage, or left hemisphere dominance. The observed swap of hemisphere dominance would not occur when the lexical tone was substituted with a meaningless pure tone. This swap reveals dependence of hemisphere labor division initially on acoustic and then on functional cues of auditory inputs in the processing from sound to meaning.

## Introduction

It is believed that human beings heard sounds that were originally neither music nor language–a protolanguage, and then divided dichotomously. The music component tuned more for emotion while the speech component extracted meaning for communication (Masataka, 2009). We now know that the human brain also becomes specialized with music preferentially processed in the right hemisphere and speech in the left (Mostafa et al., 1989; Obleser et al., 2007). However, the question of which cues the brain uses to determine this labor division remains unresolved and debated. The functional hypothesis and acoustic hypothesis are two particularly salient hypotheses that attempt to account for this mystery. The functional hypothesis claims that rightward music and leftward speech processing are due to the distinct functional domains of music and speech (Whalen and Liberman, 1987; Liberman and Whalen, 2000). The acoustic hypothesis, on the other hand, claims that the processing of tone-like sounds, such as music, is lateralized to the right hemisphere due to the slow changes in spectral properties, and that of speech is lateralized to the left due to the rapid changes in temporal properties (Zatorre and Belin, 2001; Zatorre et al., 2002). Neither of these two competing hypotheses can explain the full range of experimental data (Shankweiler and Studdert-Kennedy, 1967, 1975; Shtyrov et al., 2000).

Hemisphere lateralization in the auditory processing of tonal languages makes an interesting case for testing the acoustic hypothesis and the functional hypothesis. Tonal languages, such as Mandarin Chinese, use lexical tones along with consonants and vowels to define word meaning. Syllable/bai/in Mandarin Chinese, for example, can be accented in four lexical tones /bai1/, /bai2/, /bai3/, and /bai4/ to represent four completely different auditory words that mean “split,” “white,” “swing,” and “defeat,” respectively. So, a lexical tone is actually a semantically meaningful linguistic pitch. The semantic function and the tonal nature of lexical tones make them ideal auditory materials for testing whether the functional hypothesis or the acoustic hypothesis is true with regard to which cues are used by the brain for hemisphere lateralization in auditory cognitive processing (Zatorre and Gandour, 2008). The functional hypothesis will predict left hemisphere lateralization for processing lexical tones due to their linguistic function. In contrast, the acoustic hypothesis will predict right hemisphere lateralization for processing lexical tones due to their spectral features.

**FIGURE 1:**
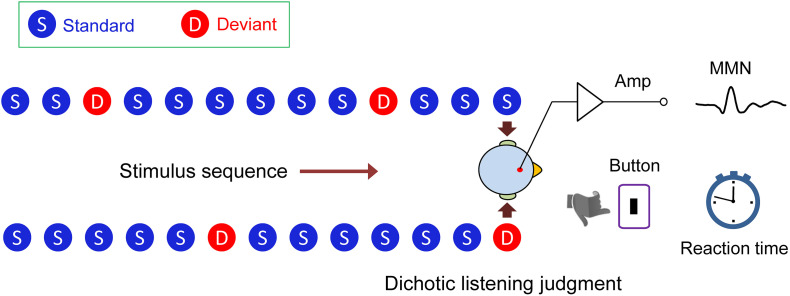
Dichotic listening oddball paradigm. In the lexical tone experiment, the standard stimulus was a sound (bai1) (syllable/bai/with a flat tone) to both ears (diotic sounds). Occasionally, the sound/bai1/to one of the ears was replaced by /bai2/, or /bai3/, or /bai4/ (syllable/bai/ with a rising, or a dipping, or a falling tone) as the deviant stimulus (dichotic sounds). In the pure tone experiment, the standard stimulus was a 550 Hz pure tone and the deviant stimulus was a 750 or 350 Hz pure tone. Mismatch negativity response (MMN) and reaction time during dichotic listening judgment were simultaneously recorded.

We designed a novel dichotic listening oddball paradigm (Figure 1). Using this paradigm, we were able to simultaneously measure the mismatch negativity (MMN) brain response, which indexes auditory processing at an early, preattentive stage, and the behavioral reaction time, which reflects auditory processing at a later stage, while the subject performed a task of dichotic listening judgment for interpreting semantic meaning of a lexical tone. We actually found a swap of hemisphere dominance from right to left during the auditory cognitive processing of Chinese lexical tones in native speakers, demonstrating that the acoustic hypothesis and the functional hypothesis are not mutually exclusive, but that each holds at a different temporal stage of processing.

## Materials and Methods

### Subjects

Twenty-one native speakers of Mandarin Chinese (11 females and 10 males, age range = 20–26 years, university students) participated in the study. All the subjects were from University of Science and Technology of China and had normal hearing. They were musically untrained and right-handed with no history of neurological or psychiatric impairment. The protocols and experimental procedures employed in this study were reviewed and approved by the Biomedical Research Ethics Committee of University of Science and Technology of China. An informed written consent was obtained from each subject.

### Stimuli

The stimuli used in the present study were adapted from our previous study (Luo et al., 2006) in which Mandarin consonant-vowel (CV) syllables /bai1/, /bai2/, /bai3/, and /bai4/ were employed. The stimuli were originally pronounced by an adult male Mandarin speaker (Sinica Corpus, Institute of Linguistics, Chinese Academy of Social Sciences, Beijing, China), and were normalized to be 350 ms, including 5-ms linear rise and fall time. The fundamental frequency (F0) contour was modified to be flat, rising, dipping, and falling to generate the four tones, using Praat software (Institute of Phonetic Sciences, University of Amsterdam, Netherlands). We then extracted these F0 contours and synthesized new syllables from the syllable/bai1/. By this means, the flat F0 contour was replaced by the rising, dipping, and falling contour, and we got four syllables with four tones, i.e., bai1, bai2, bai3, and bai4. The dichotic and diotic stimuli used in the present study were converted from monophonic stimuli. The dichotic stimuli were defined as simultaneously presented two non-identical stimuli to the left and right ear, whereas the diotic stimuli consisted of two identical stimuli presented to both ears. We first generated the diotic stimuli (bai1–bai1). Then syllable/bai1/in one channel (left or right) was replaced by either /bai2/, /bai3/, or/bai4/, and by this means we obtained six dichotic stimuli: (bai1–bai2), (bai1–bai3), (bai1–bai4), (bai2–bai1), (bai3–bai1), and (bai4–bai1). We also used the same method to create pure tone diotic and dichotic stimuli. Three monophonic pure tone stimuli were generated with frequencies at 350, 550, and 750 Hz and with a 200 ms duration including 5-ms linear rise and fall time. The 550 Hz pure tone was converted to diotic stimuli (550–550 Hz). The 550 Hz pure tone in one channel was replaced by either a 350 Hz or a 750 Hz pure tone to generate four dichotic pure tone stimuli: (550–350 Hz), (550–750 Hz), (350–550 Hz), and (750–550 Hz).

### Procedure

The stimuli were presented in an oddball manner with the diotic stimuli (bai1–bai1) as the standard, and the dichotic stimuli (bai1–bai2), (bai1–bai3), (bai1–bai4), (bai2–bai1), (bai3–bai1), and (bai4–bai1) as the deviant. The stimulus sequence was presented either in a passive condition in which participants were instructed to ignore the sounds and watch a silent movie, or in an attentive condition in which participants were required to focus on the sound and respond to deviant stimuli. Participants responded to deviant tones 2, 3, or 4 presented in either the left or right ear by pressing one of the three buttons. The stimulus order was pseudorandom with a restriction that each deviant was separated by at least two standards. The stimulus onset asynchrony was 1 s for all conditions. The pure tone stimuli were presented in the same oddball manner as the lexical tones. In the attentive condition, participants were required to respond to the occasionally presented dichotic pure tones which were higher (750 Hz) or lower in frequency (350 Hz) than the frequently heard ones (550 Hz) in either channel, by pressing a button. The detection thresholds of speech and non-speech signals were measured first and all signals were then presented binaurally at 60 dB above the detection thresholds for each listener through headphones (TDH-39; Telephonics, Farmingdale, NY, United States) in an electrically shielded soundproof room. In the passive condition, MMN was evoked by the lexical tone (or pure tone) contrast appearing in the left or right ear. In the attentive condition, MMN brain response and reaction time during the behavioral dichotic listening judgment task were recorded simultaneously in a single paradigm, which index hemisphere dominance at an early stage and at a later stage. In both lexical tone and pure tone conditions, four blocks were presented, two for the passive recordings and two for the attentive recordings. Totally eight blocks were presented to each subject and each block consisted of 800 trials. The order of the blocks was counterbalanced across the subjects. The standard was presented with a probability of 0.75 and the deviant with a probability of 0.25 (0.0417 for each deviant type for the lexical tone contrast and 0.0625 for each deviant type for the pure tone contrast).

### Data Recording and Analysis

Electroencephalogram (EEG) was recorded (SynAmps 2, NeuroScan) with a cap carrying 64 Ag/AgCl electrodes placed at standard locations covering the whole scalp (the extended international 10–20 system). Electrical activities from left mastoid (LM) and right mastoid (RM) were recorded using additional two electrodes. The reference electrode was attached to the nasal tip and the ground electrode was placed on the forehead. Vertical electrooculogram (EOG) was recorded using bipolar channel placed above and below the left eye, and horizontal EOG lateral to the outer canthi of both eyes. Vertical EOG artifacts were corrected using a regression-based procedure. Electrode impedances were kept <5 kΩ. Alternating current signals were filtered on-line with a band-pass of 0.05–100 Hz and sampled at a rate of 500 Hz. The recorded data were off-line low-pass (25 Hz) filtered with a finite impulse response (FIR) filter. Epochs obtained from the continuous data and rejected with fluctuation in amplitude >75 μV, were 800 ms in length, including a 100 ms pre-stimulus baseline. The ERPs evoked by standard and deviant stimuli were calculated by averaging individual trials. The time zero was defined as the onset of stimuli. On average, the artifact rejection removed ∼12% of the deviant trials for the passive condition, and ∼20% of the deviant trials for the attentive condition. The numbers of deviant stimuli presented in left and right ear after the artifact rejection were equal in both passive (mean 176 vs. 172 for lexical tone and 169 vs. 174 for pure tone) and attentive conditions (mean 155 vs. 159 for lexical tone and 159 vs. 162 for pure tone). The MMN waveforms were obtained by subtracting the ERP response to the standards from that to the deviants. To evaluate the effect of hemisphere dominance, we calculated mean amplitudes of MMN recorded from five electrodes (AF3, F3, FC3, F5, and FC5) over the left side of the scalp and five electrodes (AF4, F4, FC4, F6, and FC6) over the right. These responses were then averaged within a 60-ms time window ranging from 30 ms before and after the peak latency of MMN for each participant. MMN response amplitude was analyzed using a two-way ANOVA with tone status (lexical tone and pure tone) and hemisphere (left and right) as with-subject factors. For the behavioral data, the reaction time for each deviant stimulus was analyzed. To evaluate the laterality effect on the behavioral dichotic listening, we compared the reaction time for the deviant tones presented in the right ear with those in the left. All the lateralized effects were calculated using one-way RM ANOVA (within group).

Measure the mismatch negativity source analysis was performed with BESA Research software (v.5.3.7, MEGIS Software GmbH, Munich, Germany). We first computed a 3D SCD topographic mapping with the grand average MMN. The SCD maps, expressed in μV/cm^2^, were constructed by computing second spatial derivatives (the Laplacian) of the scalp field potentials. This method reduces the effects of volume conduction to the scalp potential and allows for better visualization of the approximate locations of intracranial generators that contribute to MMN. We then conducted a local auto regressive average (LAURA) distributed linear inverse solution at the peak of global field power (GFP) of MMN waveform using a lead field (solution space) with the value of regularization of 0.03%. LAURA depicts the degree of SCD brain activity within derived source regions, which allows us to show the source of MMN located in the left and right auditory cortex. To further measure the MMN source strength, we obtained equivalent current dipoles for each subject using a realistic head model with conductivity ratio of 60, and compared the dipole strength in the left and right auditory cortex. The dipole was analyzed by applying a two-dipole model with a constraint that the two dipoles were symmetrically located in the left and right hemispheres (mirror dipoles), in order to compare the dipole movement between the two hemispheres. The dipole location and orientation were fitted at the peak of GFP of MMN waveform and the dipole moments in two hemispheres were obtained. Principal component analysis decomposition showed that the two dipole sources located in the left and right auditory cortex would sufficiently explain the majority of the variance in the MMN waveform, with a goodness of fit ranging from 85.4 to 99.6% (mean = 95.5%). The displayed LAURA source and dipoles were superimposed to the BESA standard MRI.

## Results

**FIGURE 2:**
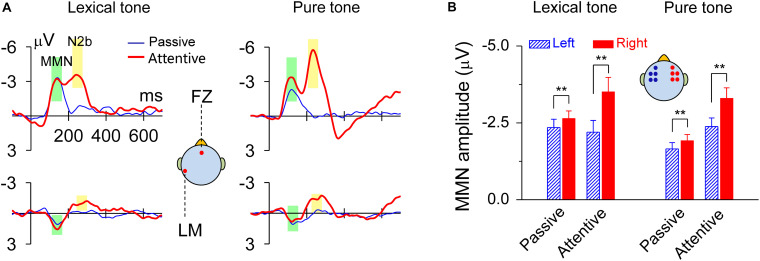
MMN responses to lexical tone contrasts or to pure tone contrasts were lateralized to the right scalp. **(A)** Grand average traces of MMN in response to lexical tone and pure tone contrasts at FZ and LM (left mastoid) electrodes. The data are band-pass filtered (1–25 Hz) for graphic illustration only. **(B)** Statistics of average MMN responses from five electrodes (AF3, F3, FC3, F5, and FC5) on the left scalp and five (AF4, F4, FC4, F6, and FC6) on the right scalp. Asterisks indicate a statistically significant difference. ^∗^*P* < 0.05; ^∗∗^*P* < 0.01. Vertical lines represent one standard error.

The MMN responses to lexical tones and pure tones recorded during the dichotic listening judgment, as well as in the passive condition, showed maximal amplitudes in fronto-central sites and a reversed polarity at mastoid sites. There was also an N2b component subsequent to MMN during the dichotic listening judgment when subjects attended to the sounds (Figure 2A). The peak latency of MMN elicited by the lexical contrast was 140 ms in both the passive condition and the attentive condition. The peak latency of MMN elicited by the pure tone contrast was 120 ms in both the passive condition and the attentive condition. When compared the mean amplitudes of the standard ERP and the deviant ERP within a 60-ms time window ranging from 30 ms before and after the peak latency (140 ms for the lexical tone and 120 ms for the pure tone), all *Ps* = 0, indicating that the MMNs elicited in all conditions were very robust (Figure 2). MMN response amplitudes were analyzed by using a two-way ANOVA with tone status (lexical tone and pure tone) and hemisphere (left and right) as within-subject factors, for attentive and passive conditions, respectively. For the attentive condition, the results showed only a significant main effect for hemisphere [*F*(1, 20) = 20.67, *P* < 0.01], indicating the MMNs were right hemisphere lateralized. There was no significant main effect for tone status [*F*(1, 20) = 0.00, *P* > 0.9] and no interactions between the two factors [*F*(1, 20) = 0.85, *P* > 0.3]. One-way RM ANOVA with hemisphere (left and right) as the factor was performed for each tone type. The results showed that MMN response amplitude was greater over the right scalp than over the left for both the lexical tone contrast [*F*(1, 20) = 10.83, *P* < 0.01] and pure tone contrast [*F*(1, 20) = 15.90, *P* < 0.01] (Figure 2B and Supplementary Figure 1A).

For the passive condition, the results showed a significant main effect for hemisphere [*F*(1, 20) = 20.38, *P* < 0.01], indicating the MMNs were right hemisphere lateralized; and a significant main effect for tone status [*F*(1, 20) = 5.81, *P* < 0.05], indicating that MMN response to lexical tones differed with that to pure tones in amplitude. There were no interactions between the two factors [*F*(1, 20) = 0.05, *P* > 0.8]. One-way RM ANOVA with hemisphere (left and right) as the factor was performed for each tone type. The results also showed that MMN response amplitude was greater over the right scalp than over the left for both the lexical tone contrast [*F*(1, 20) = 10.92, *P* < 0.01] and pure tone contrast [*F*(1, 20) = 10.30, *P* < 0.01] (Figure 2B and Supplementary Figure 1B). This finding was also confirmed in a supplementary experiment, in which diotic stimuli /bai1/ and /bai2/ served as the standard and deviant stimuli in one block, and exchanged their status in the other block. MMN was hence obtained from physically identical stimuli and showed the same patterns of hemisphere dominance (Supplementary Figure 2).

**FIGURE 3:**
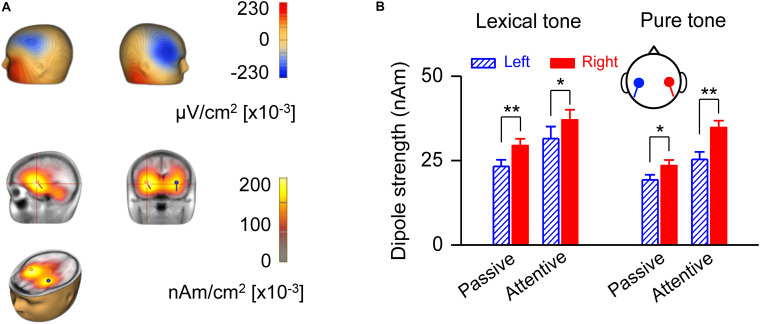
Analysis of MMN source strength in the two hemispheres. **(A)** Scalp current density topography at the peak of global field power of grand-averaged MMN in response to lexical tone contrasts under the attentive condition (upper panel). Source localization estimated by Local Auto Regressive Average and dipole solution of MMN in response to lexical tone contrasts under attentive condition (lower panel). **(B)** Average dipole strengths obtained from dipole solutions for individual subjects. Asterisks indicate a statistically significant difference. ^∗^*P* < 0.05; ^∗∗^*P* < 0.01. Vertical lines represent one standard error.

A source strength analysis of the MMN responses confirmed right hemisphere dominance and revealed possible locations of neural generators for early processing of lexical tones (Figure 3). The scalp current density (SCD) showed on the scalp surface a negative polarity over the frontocentral site and a positive polarity around the inferotemporal site in the attentive condition (Figure 3A, upper panel), indicating bilateral temporal generators accounting for MMN responses to lexical tones. LAURA, a distributed source analysis, and dipole solution, a discrete source analysis, further confirmed that the generators of the MMN are located in the left and right temporal cortex in the attentive condition (Figure 3A, lower panel). The dipole strength indicated right hemisphere dominance of MMN in response to lexical tones under both passive [*F*(1, 20) = 33.35, *P* < 0.01] and attentive [*F*(1, 20) = 4.71, *P* < 0.05] conditions. The dipole strength of MMN in response to pure tone contrasts also indicated right hemisphere dominance under both passive [*F*(1, 20) = 7.40, *P* < 0.05] and attentive [*F*(1, 20) = 11.90, *P* < 0.01] conditions (Figure 3B).

**FIGURE 4:**
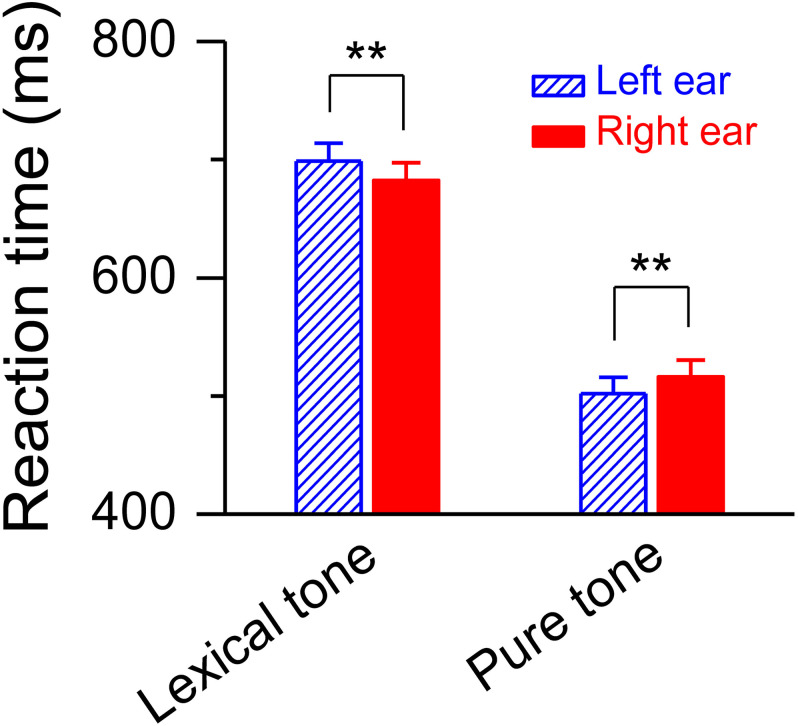
Reaction time for dichotic listening judgment. Statistics showing shorter reaction time to the deviant lexical tone, but longer reaction time to the deviant pure tone, in the right ear than in the left ear during the dichotic listening judgment task. Asterisks indicate a statistically significant difference. ^∗∗^*P* < 0.01. Vertical lines represent one standard error.

The simultaneously recorded reaction time during the dichotic listening judgment was shorter for the deviant lexical tone that presented in the right ear than in the left [682.59 ± 14.78 ms vs. 698.71 ± 15.05 ms, *F*(1, 20) = 18.79, *P* < 0.01] (Figure 4), demonstrating a right ear listening advantage, or left hemisphere dominance, at a later stage of lexical tone processing. This swap of hemisphere dominance from right to left reveals an acoustically modulated mechanism at an early stage and a functionally modulated mechanism at a later stage in auditory cognitive processing of meaning from sound. To further test the two hypotheses, we simultaneously recorded the MMN response and the reaction time to a pure tone contrast while the subjects performed a task for pitch level judgment of the pure tone contrast using the same dichotic listening oddball paradigm. A pure tone contrast shares acoustical similarities with a lexical tone contrast and is also a difference in pitch, but does not carry any semantic information. Thus, both functional and acoustic hypotheses will predict right hemisphere dominance for auditory cognitive processing of a pure tone. Our results were consistent with this prediction. The simultaneously recorded reaction time during the dichotic listening judgment was shorter for the pure tone contrast appearing in the left ear than in the right [501.99 ± 13.84 ms vs. 516.10 ± 14.14 ms, *F*(1, 20) = 12.25, *P* < 0.01] (Figure 4), demonstrating a left ear listening advantage, or right hemisphere dominance, at a later stage of processing. These results indicate that a swap of hemisphere dominance does not occur during auditory cognitive processing of a pure tone, which does not have a semantic function.

## Discussion

The novel dichotic listening oddball paradigm employed in the present study enabled us to explore the hemisphere dominance for auditory cognitive processing at two distinct temporal stages in a single paradigm. The MMN brain response, an index of early auditory processing, to lexical tone contrast is lateralized to the right hemisphere, which agrees with the acoustic hypothesis. The simultaneously recorded reaction time during the dichotic listening judgment, an indicator of later auditory processing, indicates left hemisphere dominance, which agrees with the functional hypothesis. Consistent dichotic listening judgment can also be found in a recent study (Mei et al., 2020), showing LEA in the acoustic analysis of suprasegmental information of tones and REA in the phonological and lexical-semantic processes of tones. Our results demonstrate that the acoustic hypothesis is true at an early stage of auditory processing whereas the functional hypothesis is true at a later stage of auditory processing, which has been speculated by some investigators (Zatorre et al., 2002; Mei et al., 2020), but unproved until now. The swap of hemisphere dominance reveals dependence of hemisphere labor division initially on acoustic and then on functional cues of auditory inputs in the processing of meaning from sound.

We found that attention did not change the hemisphere dominance in the early auditory processing of lexical tones and pure tones (Figures 2, 3). But this does not mean that top-down control or language experience has no influence on low-level auditory processing. Evidence has shown that language experience facilitates the encoding of linguistic pitch patterns as early as at the brainstem level (Krishnan et al., 2011). For early auditory processing, our proposal of acoustic dominant mechanism does not conflict with the experience-dependent mechanism, because language experience can enhance the sensitivity to acoustic dimensions of the sounds that one is familiar with whereas hemisphere dominance still mainly depends on acoustic properties of input sounds. We found right-hemisphere lateralized MMN based on greater MMN amplitude over the right scalp than over the left. The MMN dipole strength comparison was a supplementary analysis because source localization involved an inverse problem and provided relatively poor spatial resolution. To compare dipole strength in the two hemispheres, we used a two-dipole model which was in line with previous MMN studies (Naatanen et al., 1997; Luo et al., 2006; Yasui et al., 2009; Wang et al., 2012, 2013). The mirror dipole method we deployed in the present study has been shown to be effective in the comparison of the dipole strengths between the two hemispheres (Luo et al., 2006; Wang et al., 2012, 2013), when anatomical data of individual subject was not available.

The swap of hemisphere dominance in lexical tone processing may manifest an evolutionary path of the human brain: All the animals have neural structures for the universal task of extracting acoustic attributes of a sound, but the human has evolved a linguistic center on top of these structures for the unique task of decoding semantic meaning from the extracted acoustic attributes. For the human brain, and perhaps also for some animal brains, labor division between the two hemispheres is based on the acoustic attributes of auditory inputs in low level cognitive processing. For the higher level cognitive task of extracting semantic meaning from lexical tones executed by native speakers, the linguistic center in the left hemisphere is destined to be recruited following recruitment of the right hemisphere for analyzing acoustic attributes, causing a swap of hemisphere dominance from right to left during processing. This swap is not predicted to happen in non-native speakers with no tonal language experience or in animals.

## Data Availability Statement

The original contributions presented in the study are included in the article/Supplementary Material, further inquiries can be directed to the corresponding author/s.

## Ethics Statement

The studies involving human participants were reviewed and approved by Biomedical Research Ethics Committee, University of Science and Technology of China. The patients/participants provided their written informed consent to participate in this study.

## Author Contributions

X-DW, LC, and H-WL: theoretical proposition and experimental design. HX, X-DW, and ZY: experimental methods, data collection, and data processing. X-DW, LC, MW, and H-WL: manuscript preparation. All authors contributed to the article and approved the submitted version of the manuscript.

## Conflict of Interest

The authors declare that the research was conducted in the absence of any commercial or financial relationships that could be construed as a potential conflict of interest.
