# Supplementary material for: Brain Hemispheres Swap Dominance for Processing Semantically Meaningful Pitch
Source: Front Hum Neurosci. 2021 Feb 3;15:621677. doi: 10.3389/fnhum.2021.621677 (PMC7886982; doi:10.3389/fnhum.2021.621677)
Supplement: Supplementary file 1 [file Data_Sheet_1.PDF]

Supplementary Information for:

Brain Hemispheres Swap Dominance for Processing Semantically Meaningful Pitch

Xiao-Dong Wang[1,2], Hong Xu[3], Zhen Yuan[4], Hao Luo[5], Ming Wang[2], Hua-Wei Li[6*] & Lin Chen[2*]

[1]Faculty of Psychology, Southwest University, Chongqing, China

[2] Auditory Research Laboratory, School of Life Sciences, University of Science and Technology of China, Hefei 230027, China

[3]Division of Psychology, Nanyang Technological University, Singapore

[4]Bioimaging Core, Faculty of Health Sciences, University of Macau, Macau SAR, China

[5]Department of Otolaryngology-Head and Neck Surgery, Wayne State University School of Medicine, Detroit, USA.

[6]Affiliated Eye and ENT Hospital,Fudan University, Shanghai 200031, China

*Correspondence and requests for materials should be addressed to

Lin Chen, email: linchen@ustc.edu.cn

Hua-Wei Li, email: hwli@shmu.edu.cn

S1. Event-related potential (ERP) in response to lexical tone stimuli (standard and deviant) and topographic maps of the difference wave

The ERPs in response to the standard and deviant lexical tone stimuli were meaured for both attentive and passive conditions, respectively. We constructed the topographic maps at the peak latency of the difference waves. The MMN response to lexical tones showed fronto-central topography with right hemisphere dominance for both attentive and passive conditions. For the attentive condition, there was also an N2b component subsequent to MMN with its maximum occurring at the centro-parietal sites and with no polarity reversal at the mastoid sites (Fig. 2a). This is because the N2b generators were widespread and located outside the auditory cortex[1]. We investigated the hemisphere dominance of MMN by calculating mean amplitudes of MMN recorded from five electrodes (AF3, F3, FC3, F5 and FC5) over the left side of the scalp and from five ones (AF4, F4, FC4, F6 and FC6) over the right, as shown in Fig. 2b. Here, we also compared the mean amplitude of deviant ERPs (at the same time window as for MMN) over the left and right scalp directly using the same method. The deviant lexical tone elicited larger response over the right scalp in both passive (t_{20} = 2.29, P < 0.05) and attentive (t_{20} = 2.94, P < 0.01) conditions.

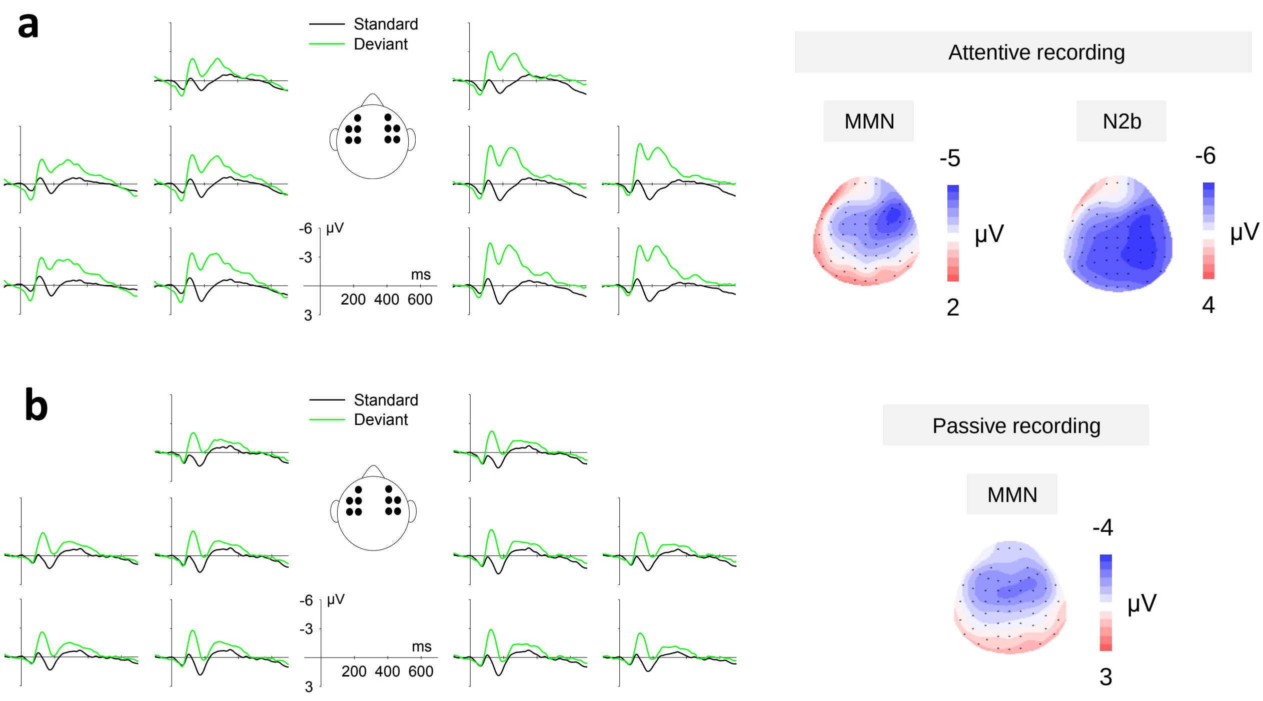

Figure S1 | ERP responses to standard and deviant lexical tone stimuli. Grand-averaged ERPs for the standard and the deviant in an attentive condition (a) and in a passive condition (b) recorded at AF3, AF4, F3, F4, FC3, FC4, F5, F6, FC5 and FC6 sites. A 2-D topographic map (right panels) was constructed for the MMN and N2b components at their peak latencies.

S2. Hemisphere dominance in lexical tone processing as revealed by using a deviant-standard-reverse paradigm

We tested another group of eight subjects on the MMN response to lexical tones using a deviant-standard-reverse paradigm. In one block, syllable /bai1/ was presented frequently as the standard stimuli (85%) and syllable /bai2/ was presented infrequently as the deviant stimuli (15%). In the other block, the syllable /bai2/ was presented as the standard whereas /bai1/ as the deviant. The stimuli were diotically presented. Each block consisted of 1000 trials and was run once. The other parameters were the same as those in the main experiment. The MMN response to syllable /bai1/ was derived by comparing the ERPs in response to /bai1/ when it served as the deviant (in one block) and the standard (in the other block). The MMN response to syllable /bai2/ was derived in the same manner. The MMNs in response to /bai1/ and /bai2/ showed similar right-hemisphere weighted topographies. ERP waveforms for physically identical standard and deviant stimuli at Fz, FCz, F3, FC3, F4, FC4, F5, FC5, F6 and FC6 sites were shown in Fig. S2. We evaluated the hemisphere dominance for the MMN response by comparing mean amplitudes of MMN recorded from five electrodes over the left side of the scalp (AF3, F3, FC3, F5 and FC5) with those over the right (AF4, F4, FC4, F6 and FC6). The results showed a right hemisphere dominance for the MMN response to both /bai1/ and /bai2/ ($t_7 = 3.02$, $P < 0.05$ and $t_7 = 4.27$, $P < 0.01$).

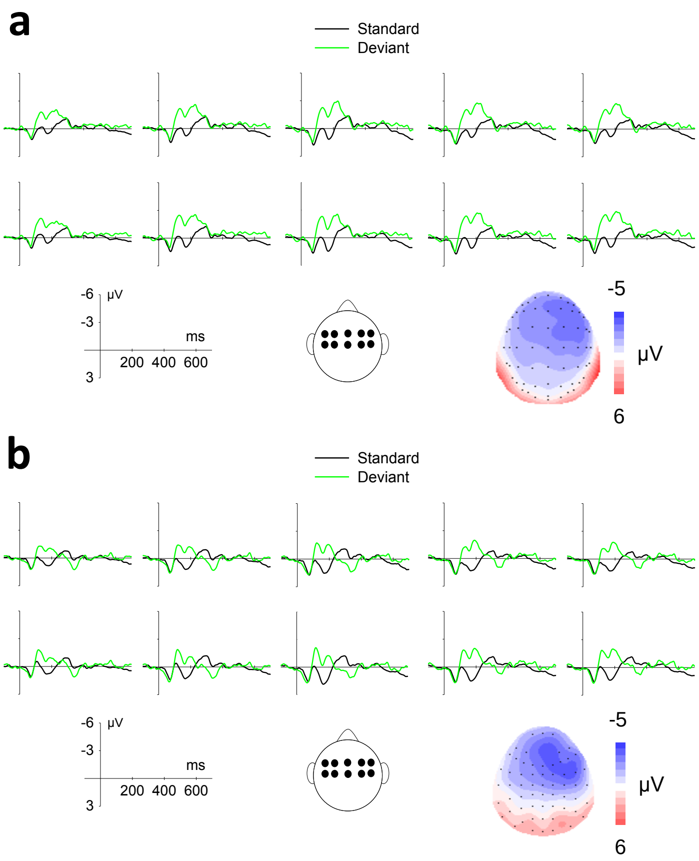

Figure S2 | ERP responses to the lexical tone stimulus serving as the standard or the deviant. (a) Grand-averaged ERPs in response to syllable /bai1/ when it served as the standard and deviant in separate blocks (n = 8). (b) Grand-averaged ERPs in response to syllable /bai2/ when it served as the standard and deviant in separate blocks (n = 8). Topographic maps of MMN were constructed by subtracting the ERP response to /bai1/ (or /bai2/) serving as the standard in one block from that serving as the deviant in the other block.

Reference

1 Woldorff, M. G., Hillyard, S. A., Gallen, C. C., Hampson, S. R. & Bloom, F. E. Magnetoencephalographic recordings demonstrate attentional modulation of mismatch-related neural activity in human auditory cortex. *Psychophysiology* **35**, 283-292 (1998).
